# Vaccination in Italy

**Published:** 1890-05-10

**Authors:** 


### VACCINATION IN ITALY.

THERE is no compulsory vaccination in Lombardy, and no penalty of fine or imprisonment. Practically compulsion, however, is brought to bear indirectly, for no pupil is admitted to school unless he presents a certificate of vaccination, no employé into service, and no recruit to the army. A very serious epidemic of small-pox raged in Milan some years ago, and was attributed by medical men to the difficulty of reaching the masses of the poor in the lower quarters of the town and compelling vaccination. Vaccination is always effected with calf-lymph, never from arm to arm.

